# The impact of integrating medical oxygen indicators into DHIS-2 on the reporting of hypoxaemia diagnosis and management: the case of Cameroon

**DOI:** 10.7189/jogh.15.04088

**Published:** 2025-02-14

**Authors:** Yauba Saidu, Clarence Mbanga, Ngassa Andinwoh, Andreas Frambo, Ousmane Diaby, Rogers Ajeh, Audrey Battu, Zakary Katz

**Affiliations:** 1 Clinton Health Access Initiative, HS Jean-Paul II Boulevard, Tsinga sous-prefecture, Yaoundé, Cameroon; 2 Institute for Global Health, Santa Chiara Lab, University of Siena, Siena, Italy; 3 College of Public Health and Health Professions, University of Florida, Florida, USA; 4 Faculty of Economics and Management, University of Ngaoundere, Cameroon; 5 HIV, TB, and Malaria Grant Coordination Unit, Ministry of Public Health, Yaoundé, Cameroon; 6 Global Essential Medicines, Clinton Health Access Initiative Inc., Boston, Massachusetts, USA

## Abstract

**Background:**

Between 2021 and 2023, the Cameroon Ministry of Public Health, with support from the Clinton Health Access Initiative (CHAI), made considerable investments in establishing a reliable medical oxygen system in Cameroon. To monitor the impact of said investments, medical oxygen indicators were identified and integrated into the country’s health information management system. This integration aimed to enhance the collection, reporting, and analysis of medical oxygen data, ultimately improving decision-making regarding oxygen needs, procurement volumes, and patient referrals based on real-time data on the availability of oxygen supplies. Here we outline the integration approach and assess its impact on medical oxygen reporting one year post-investment.

**Methods:**

We adopted an iterative, consultative approach involving multiple meetings and workshops with all key stakeholders to define medical oxygen indicators and their technical specifications, develop the necessary data collection forms and guides, pre-test the defined indicators, review and validate them, and finally integrate them into the District Health Information System 2 (DHIS-2). Following integration, we rolled out the indicators within DHIS-2 nationwide using a two-step process, beginning with cascaded training of regional- and district-level data managers on the reporting of medical oxygen indicators into DHIS-2, and followed by supervision and mentoring. We assessed the impact of this rollout by comparing reporting rates on medical oxygen use before and after the integration and training process.

**Results:**

We validated 15 indicators and integrated them into the DHIS-2, and we trained 218 regional- and district-level data managers from eight of the country’s ten regions on leveraging the defined indicators to capture data on medical oxygen use and hypoxaemia management at the facility and input it into the system. We observed a 23% absolute increase in the completeness of medical oxygen reports, with rates rising from 3% in December 2022 (pre-intervention) to 26.2% in December 2023 (one year post-intervention). We also noted a considerable increase in the reporting of case management, with, for instance, the number of newborns diagnosed with hypoxaemia rising from zero pre-integration and training to 213 by March 2024.

**Conclusions:**

Integration of medical oxygen indicators into DHIS-2, along with staff training, improved reporting rates for medical oxygen use and hypoxaemia management. Continuous support and infrastructure upgrades are needed to sustain investment.

Hypoxaemia, a condition resulting from a wide array of medical and surgical states and characterised by abnormally low levels of blood oxygen levels, remains a significant contributor to disability and mortality in low- and middle-income countries (LMIC), particularly among vulnerable populations such as mothers, newborns, and children. Curbing this burden requires timely diagnosis and management through scaling up interventions like pulse oximetry for the diagnosis and medical oxygen for the management of the condition in health facilities [1]. Such interventions have been shown to drastically improve health outcomes of patients with hypoxaemia [2].

In recognition of the importance of medical oxygen in reducing the morbidity and mortality associated with hypoxaemia, and in alignment with its commitment to reducing maternal, neonatal, and child mortality, the Cameroon government, via its Ministry of Public Health (MoPH), earmarked the improvement of medical oxygen availability and accessibility as one of its key priorities in its 2016–2027 Health Sector Strategy. The dire need to establish reliable medical oxygen systems was further highlighted by the COVID-19 pandemic, which exacerbated demand for medical oxygen [3]. Despite its critical importance, proven benefits, and classification as an essential medicine by the World Health Organization [3], the availability and accessibility of medical oxygen has remained a long-standing challenge in Cameroon (as is the case with most sub-Saharan African countries), impeded by gaps that span across the health system, including the availability of biomedical equipment and supplies; facility infrastructure; availability of trained health personnel and biomedical personnel; comprehensive national strategy and sustainable financing; and data utilisation for decision-making.

Cameroon, like many other LMICs, faces considerable challenges in accurately measuring and monitoring medical oxygen use – a challenge that is partly linked to fragmented data management systems [4]. Data management systems in most LMICs, particularly in sub-Saharan Africa, are dominated by paper-based systems that tend to generate incomplete and inaccurate reports [5]. Despite this longstanding challenge, evidence from numerous studies shows that an enhanced and standardised health reporting system plays a key role in improving healthcare systems, as it facilitates the generation of timely and reliable information for effective planning, monitoring, and evaluation of service delivery across all of their levels [5–7].

In recent efforts to develop a national strategic plan for the provision of medical oxygen in Cameroon, we conducted a rapid situational analysis to identify and characterise gaps in the medical oxygen ecosystem in Cameroon [8]. Some of the key bottlenecks we observed included the lack of policies and guidelines on the use of medical oxygen; insufficient availability of equipment, materials, and consumables for the diagnosis and management of hypoxaemia; insufficient funding for medical oxygen; and inadequate systems for collecting and transmitting data on the use of medical oxygen. Of the 114 health facilities surveyed, only 15% reported documenting data on hypoxaemia management with medical oxygen. These facilities, in turn, did not collect or report data in a standardised manner, as the national health information management system (*i.e.* the District Health Information System 2 (DHIS-2)) did not specifically capture data on hypoxaemia management and medical oxygen use. This follows prior observations from most sub-Saharan African countries, where medical oxygen use is currently not a standard health system indicator, as vaccination or bed net coverage are [9]. This is worrisome because the collection and transmission of data on medical oxygen use is critical to monitoring the progress of oxygen use and informing planning and decision-making at all levels of the health system [10]. For this reason, building systems that can ensure real-time generation and utilisation of data for decisions at the operational and national levels may be essential in helping predict and prevent outages in medical oxygen, which in turn may ensure a steady supply of medical oxygen to areas with the greatest need of this lifesaving intervention.

In response to the challenges outlined in the situational analysis, we, alongside the Clinton Health Access Initiative (CHAI) and with funding from Unitaid, provided technical support to the Cameroonian MoPH between 2021 and 2023 to lay the groundwork required to establish a reliable and sustainable medical oxygen system that could ensure uninterrupted access to medical oxygen in health facilities across the country. Specifically, we developed a governance framework for medical oxygen production and use in Cameroon, quantified the medical oxygen needs of the country to guide future investments, and set up additional systems (integration of medical oxygen indicators into DHIS-2) to enhance the collection, reporting, analysis, and use of medical oxygen data for decision making, and the monitoring of the impact of medical oxygen related investments. Here we outline the approach and results in setting up the medical oxygen data system through the integration of validated indicators into the DHIS-2 and evaluate the impact of these efforts on the reporting of medical oxygen use and hypoxaemia management one year post-investment. The lessons we learned could help inform other low-and middle-income countries, especially those in sub-Saharan Africa, that seek to implement similar projects.

## METHODS

### Study setting

We carried out this study in Cameroon, a country situated in the west-central region of Africa administratively partitioned into 10 regions, with a population of over 30 million individuals [11–13]. The healthcare infrastructure of the country is structured into three tiers: central (MoPH), intermediate (regional delegations), and peripheral (health districts) and consists of health facilities classified across six distinct categories (general hospitals, central hospitals, regional hospitals, district hospitals, medicalised health centres, and integrated health centres). In total, there were over 6000 health facilities spread across the country's 203 health districts in 2023 [14]. They were, however, unequally distributed, with the majority located in urban areas, particularly in the country's two most populous cities – Yaoundé and Douala.

### Setting up medical oxygen data systems

To ensure effectiveness, inclusivity, and government ownership, we integrated hypoxaemia management and medical oxygen use indicators into the DHIS-2 using a participatory approach and following a systematic process consisting of several successive steps.

#### Definition of oxygen indicators and their technical specification

We first developed indicators on medical oxygen use and hypoxaemia management and subsequently drove their integration into DHIS-2, intending to facilitate the routine collection, analysis, and monitoring of related data. We selected the final list of indicators approved for use based on a variety of criteria, including relevance to hypoxaemia management, ease of data capture and reporting, impact on resource planning and allocation, and alignment with national health priorities. Following their identification, we further determined the technical specifications for each indicator (specific data elements, variables, and parameters that would constitute each indicator or would be needed to compute the indicator). This process allowed for the definition of indicators that are comprehensive, accurate, and capable of capturing the most relevant information related to medical oxygen use and hypoxaemia management, to promote evidence-based decision-making.

#### Development of a data collection form and guide

Following the definition of indicators and their technical specifications, we designed a logical and user-friendly data collection form to facilitate efficient and accurate data collection. These forms collected information such as patient demographics, oxygen therapy parameters, clinical assessments, and treatment outcomes. Alongside the form, we developed a data collection guide detailing instructions and explanations, including examples and clarifications, to assist healthcare professionals in understanding and consistently implementing the data collection process. Both the form and guide were thoroughly reviewed by relevant stakeholders, including healthcare professionals, data managers, and subject matter experts. We then used their feedback to refine the tools. This iterative process resulted in a set of tools that were judged to be comprehensive, clear, and aligned with the specific needs of hypoxaemia management and medical oxygen use.

#### Pre-testing of oxygen indicators

The next step in the process involved the pre-testing of the defined indicators and data collection tools. Prior to this step, we trained staff from the selected health facilities on the target indicators, as well as the process for collecting related data and entering it into the DHIS-2. During the pre-testing phase, the trained staff collected data using the newly developed oxygen indicators, following the standardised approach and protocols provided during the training. This allowed for the assessment of the indicators' feasibility, applicability, and effectiveness in capturing oxygen-related information. We then analysed and leveraged the collected data to assess the quality of the process and identify any challenges encountered during data collection, such as technical difficulties with the data entry interface in DHIS-2. We used the feedback from facility staff on the indicators’ feasibility, applicability, and effectiveness in capturing medical oxygen use and hypoxaemia management-related information to revise and refine the indicators and data collection instruments.

#### Review and validation of indicators

We organised a workshop with stakeholders from different MoPH departments to revise the indicators and data collection tools following the pre-testing exercise. The workshop served as a platform for collaborative discussions, expert input, and critical evaluation of indicators and data collection tools, fostering in-depth discussions, comprehensive reviews, and valuable feedback to enhance their scientific rigour and applicability. By its end, a final list of indicators and data collection tools was agreed upon and transmitted to the health information unit of the MoPH for integration into DHIS-2.

#### Integration of medical oxygen indicators into DHIS-2

Following the endorsement of the final list of indicators, we organised a follow-up workshop with biomedical engineers and personnel from the health information unit to establish the technical infrastructure and workflows needed for the effective integration of the indicators into DHIS-2. Once these were defined, we reviewed the approved list of indicators and data collection tools for feasibility and practicability and subsequently used them to update the monthly activity register, further configuring them in HTML format for addition within the DHIS-2 system. We then performed a simulation of the configuration to ensure that formulae were appropriately defined and that the data prompt within DHIS-2 provided the desired indicators.

### Nationwide roll-out of medical oxygen indicators in DHIS-2

#### Capacity building of healthcare workers

With the indicators of hypoxaemia management and medical oxygen use successfully integrated into DHIS-2, the next key action was to train healthcare personnel on the capture of hypoxaemia management and medical oxygen use data, and their timely and accurate entry into the DHIS-2 system. We did this via a cascaded training approach, which we outline below.

#### Training of trainers

We trained regional-level data managers (trainers) through a three-day workshop on the reporting of hypoxaemia management and medical oxygen use data, organised in February 2023. The training aimed to equip the regional managers with the necessary skills to effectively employ the monitoring tools and dashboard on hypoxaemia management and medical oxygen use within DHIS-2 and strengthen their capacity to later train district staff from their respective regions. The workshop followed an andragogical approach, employing various methods to enhance learning, such as the administration of a pre-test to assess participant abilities, presentation of developed tools, discussions/exchanges, practical work/simulations, and a post-test to evaluate the impact of the training on the participants abilities. We facilitated the training alongside the MoPH and the United Nations Children’s Fund (UNICEF).

#### Training of district personnel

Following the training of regional data managers (trainers), we organised another three-day training workshop in March 2023 to equip district data managers and district medical officers with the skills required to capture data on hypoxaemia management use of medical oxygen, enter these data into DHIS-2, and effectively navigate the medical oxygen use dashboards within DHIS-2 to inform decision making within their respective districts. Topics covered during the training included: introduction to DHIS-2, getting started on the hypoxaemia management and medical oxygen use reporting/analysis tools in DHIS-2 (standard reports, data sets, report tables, completeness, static reports etc.) and how to use these tools for data reviews, analyses, and planning. Subsequently, we prepared a national training schedule for roll out, whereby we organised eight three-day training workshops across the supported regions (the Center, Littoral, Southwest, West, Northwest, South, East, and Adamawa regions). The Adamawa region served as a geographic representation of the North and Far North regions, which were not included in the training due to logistical challenges. These sessions were facilitated by previously trained regional data managers (trainers), under the supervision of staff from CHAI, the MoPH, and UNICEF. By June 2023, the medical oxygen reports within DHIS-2 were available for data entry at the district level.

#### Supervision and mentoring

Together with the MoPH, we jointly organised bimonthly meetings as a critical strategy for ensuring the long-term sustainability of the medical oxygen data system. These meetings served as a platform for supervisors from both organisations to assess progress, address challenges, and share best practices to improve data reporting across various health facilities and regions. During these sessions, participants reviewed the quality of submitted reports, enabling them to identify and address challenges and bottlenecks in the reporting process. Specific topics covered included the examination of common data entry errors, strategies for improving data collection, and the importance of adherence to reporting timelines. Challenges such as inconsistent reporting practices and gaps in data completeness were also addressed. For example, facilitators shared best practices from regions that had achieved higher reporting rates, highlighting techniques such as regular reminders for data submission and targeted training sessions for healthcare workers.

The joint organisation of these meetings fostered a collaborative environment and enabled a more comprehensive evaluation of reporting rates across the different levels of the health system. By cultivating an environment of ongoing mentorship and support, the bimonthly meetings contributed significantly to the sustainability of the initiative. This proactive and interactive approach helped ensure that data reporting was carried out consistently and accurately, ultimately contributing to the availability of reliable and timely data for decision-making and programme planning.

## RESULTS

We successfully validated 21 variables and 15 indicators of medical oxygen use and hypoxaemia management and integrated them into the DHIS-2 platform (**Table 1**). In addition, we trained 218 health personnel from 78 health districts across eight regions of the country on the reporting of hypoxaemia management and medical oxygen use within DHIS-2 system (**Table 2**).

**Table 1:** Hypoxaemia management and medical oxygen use indicators integrated into DHIS-2

Indicator	Variable(s) to capture
Percentage of HFs with a hypoxaemia/ hypoxia management department	Availability of a clinical department (maternity, orientation/emergency, internal medicine, intensive care unit, paediatric, *etc*.) for the management of hypoxaemia/hypoxia (yes/no)
Average number of stock-out days of medical oxygen during the quarter	Average number of stock-outs days of medical oxygen in the HF
Total quantity of medical oxygen available at the beginning of the month	Quantity of medical oxygen available in the facility at the beginning of the month
Total quantity of medical oxygen received during the month	Quantity of medical oxygen received in the facility during the month
Total quantity of medical oxygen consumed during the month	Quantity of medical oxygen consumed in the facility during the month
Total quantity of medical oxygen lost or damaged during the month	Quantity of medical oxygen lost or damaged in the facility during the month
Proportion of functioning pulse oximeters during the month	Number of functioning pulse oximeters
	Total number of oximeters available in the HF
Proportion of functioning pulse oximeters during the month	Number of functioning medical oxygen concentrators during the month
	Number of functioning medical oxygen concentrators in the HF
Proportion of 1st, 2nd, 3rd and 4th category HFs with functioning respirators	Number of 1st, 2nd, 3rd, and 4th category HFs with functioning respirators
	Number of 1st, 2nd, 3rd, and 4th category HFs with functioning respirators
Proportion of 1st, 2nd, 3rd and 4th category HFs with a functioning (pressure swing absorption/air separation unit) oxygen generator	Number of 1st, 2nd, 3rd, and 4th category HFs with a functioning oxygen generator
	Number of 1st, 2nd, 3rd and 4th category HFs with an oxygen generator
Total 7500-L medical oxygen cylinders (containing medical oxygen or not)	Number of 7500-L medicinal oxygen cylinders (containing medical oxygen or not) available in the facility
Proportion of health workers who received capacity building in hypoxia/ hypoxia management	Total number of healthcare workers trained in the management of hypoxaemia/hypoxia in the health facility during the month
	Total number of health workers in the HF
Proportion of patients affected by hypoxaemia/hypoxia who received oxygen therapy	Number of hypoxaemia/hypoxia cases treated in the HF during the month
	Number of hypoxaemia/hypoxia cases diagnosed in the HF during the month
Therapeutic success rate	Number of hypoxaemia/hypoxia cases cured in the HF during the month
	Number of hypoxaemia/hypoxia cases treated in the HF during the month
Hospital prevalence of hypoxaemia/hypoxia in the HF	Number of hypoxaemia/hypoxia cases diagnosed in the HF during the month
	Number of patients admitted in the HF during the month
Mortality rate related to hypoxaemia/hypoxia	Number of patients who died of hypoxaemia/hypoxia
	Number of hypoxaemia/hypoxia cases diagnosed in the HF during the month

HF – health facility

**Table 2:** Training of district-level staff on hypoxaemia management and medical oxygen use reporting

Region	Number of districts available	Districts trained, n (%)	Number of staff trained
Center	32	8 (25)	25
Littoral	24	12 (50)	30
South West	19	8 (42)	25
West	20	20 (100)	31
North West	20	11 (55)	22
South	12	12 (100)	27
East	15	7 (47)	22
Adamawa	12	2(17)	35
Total	154	78 (50.6)	218

### Impact of indicators integration and capacity building on reporting rates

#### Completeness of medical oxygen reports

**Figure 1:**
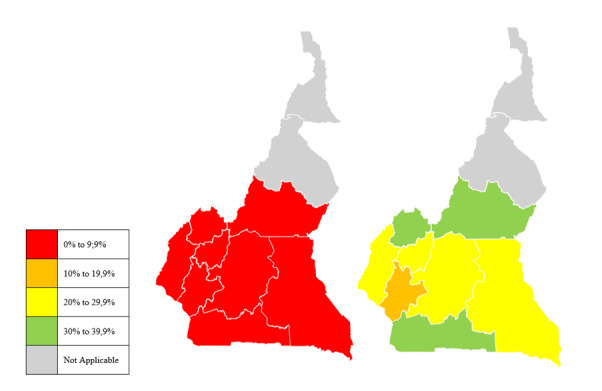
Completeness of medical oxygen reports before (December 2022) and after (December 2023) training of healthcare personnel on oxygen data reporting in DHIS-2.

**Figure 2:**
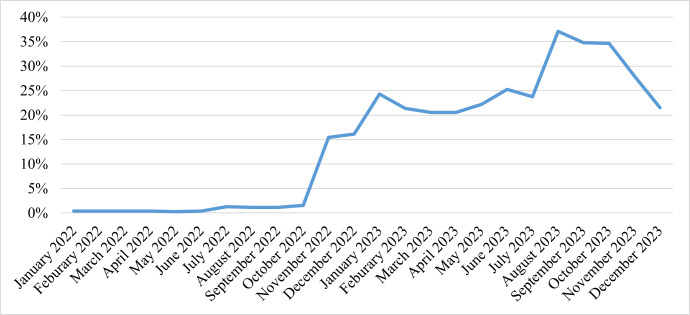
Trend in the completeness of medical oxygen reports.

There was a significant improvement in the reporting rate of hypoxaemia management-related indicators within DHIS-2 from December 2022 to December 2023 in the intervention regions whose staff were trained (**Figure 1**). More specifically, there was a steady rise in the completeness of reports on medical oxygen use and hypoxaemia management within the first 12 months following the rollout of the system, with reporting rate rising from 3% in 2022 to 26.2% in 2023 (**Figure 2**).

#### Case management

**Figure 3:**
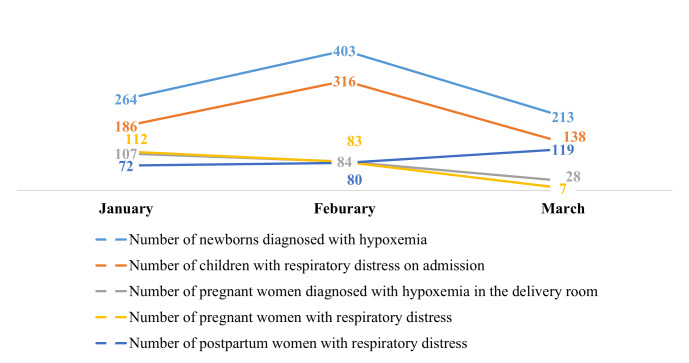
Evolution of diagnosed cases of hypoxaemia reported in DHIS-2 from January to March 2024.

**Figure 4:**
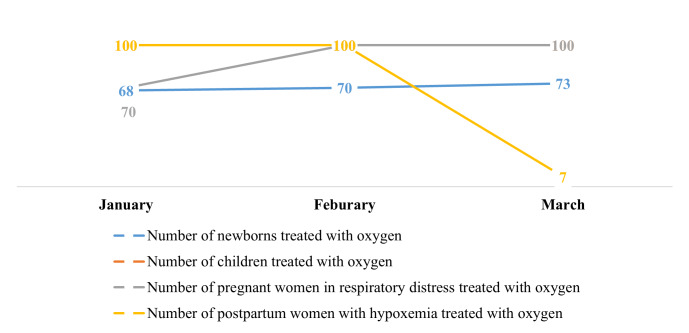
Trend in the number of reported cases of hypoxaemia or respiratory distress treated with medical oxygen between January and March 2024.

**Figure 5:**
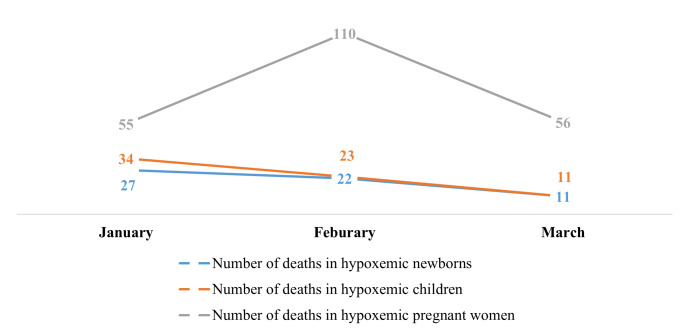
Trend in the number of reported cases of hypoxaemia deaths between January and March 2024.

There was no data reported on hypoxaemia case management prior to our integration of oxygen indicators in the DHIS-2. Health facilities only began case management reporting as from January 2024, with a total of 2217 hypoxaemia cases reported by March 2024. Despite the increase in the number of reported cases of hypoxaemia in newborns between January and February, there was an overall decrease in the number of cases reported, with values falling from 264 in January to 213 in March. A similar trend was observed for other case management indicators (**Figure 3**). Asides the number of reported cases of postpartum women with hypoxaemia that saw a decline between February and March, there was an overall improvement in the number of cases with hypoxaemia or respiratory distress treated with medical oxygen (**Figure 4**). We also observed an overall decrease in the number of hypoxaemia related deaths reported between January and March 2024 for all the different case indicators (**Figure 5**).

## DISCUSSION

Here we described the approach used in setting-up a medical oxygen data system through the integration of validated oxygen indicators into DHIS-2. Additionally, we assessed the impact of these efforts on the reporting rates of medical oxygen use and hypoxaemia management one year post-investment, highlighting insights we gained throughout the process. Overall, we configured 15 indicators and 21 variables on hypoxaemia management and medical oxygen into DHIS-2 and trained 218 district-level staff from eight of Cameroon’s 10 regions on the capture and reporting of medical oxygen use and hypoxaemia management into the system. The implemented activities considerably improved the reporting rate of hypoxaemia management within DHIS-2 in the target regions, with reporting rates increasing substantially from 3% in December 2022 to 26.2% in December 2023. There was also a notable increase in case management for hypoxaemia from the baseline in 2022.

We also observed that, despite the surge in the number of hypoxaemia cases reported in newborns between January and February, there was an overall decrease in the number of cases reported, with values falling from 264 in January to 213 in March. We observed a similar trend for other case management indicators. The reduction could be partially attributed to the positive impact of healthcare worker training, which not only enhanced their capacity for identifying and managing hypoxaemia, but also, when combined with growing familiarity with the DHIS-2 platform, likely resulted in more accurate and consistent reporting practices. As health workers gained proficiency through continued use, their accuracy and confidence in data entry may have improved, which in turn may have reduced potential inaccuracies that might have been present during the initial reporting phase. This progressive improvement, often described as ‘practice makes perfect’ [15], suggests that early training provided foundational knowledge, while ongoing experience further refined case identification and reporting skills. However, other contributing factors should also be considered. Improved case management and prevention efforts, such as early identification and timely treatment, may have reduced the progression of hypoxaemia, leading to fewer cases being reported. Additionally, seasonal factors might also play a role in the incidence of hypoxaemia cases, as environmental conditions affect respiratory health. For instance, respiratory infections often peak during specific seasons, which could influence the number of hypoxaemia cases reported [16].

Conversely, the overall increase in the reporting rate of hypoxaemia cases could be attributed to the integration of hypoxaemia management indicators into the DHIS-2 and the training of regional- and district-level data managers to capture and report hypoxaemia diagnosis and treatment data directly within this system. This not only cuts down on the bureaucratic delays commonly associated with paper-based reporting systems, but equally provides the country with a comprehensive framework for monitoring hypoxaemia management and promoting timely, evidence-based decision-making. Importantly, the integration of medical oxygen indicators into DHIS-2 had direct implications on patient care. The improved data collection processes enabled healthcare facilities to respond more quickly to medical oxygen shortages, facilitating better resource allocation and management, while the integration provided health facilities with insights on hypoxaemia case trends, allowing for the development of tailored interventions to address the situation, ultimately resulting in enhanced patient outcomes. Despite this, we did not measure the association between the improvement in reporting and other important health outcomes such as mortality from hypoxia, which is why we recommend future research to explore this association.

Despite the above limitation, the significance of the system that we set up in Cameroon cannot be overstated, particularly in light of the challenges revealed by the COVID-19 pandemic in the Cameroonian healthcare system. One such challenge was the inability of the healthcare system to effectively manage the demand and supply of medical oxygen during various surges of the pandemic. From our experience, during the peak of cases and oxygen requirements, health officials had to rely on manual, daily communication with hospitals and field staff to gather information on stock availability and usage, hindering real-time understanding of oxygen consumption and availability. This laborious process made it exceedingly difficult to make timely and informed decisions. In response, the establishment of digital systems for monitoring the availability and utilisation of medical oxygen can enable officials to track progress in site selection, construction of facilities, electrification, and the connection of oxygen pipelines to hospitals and other healthcare facilities for efficient oxygen delivery.

The positive impact of DHIS-2 on health reporting is not unique to Cameroon. Evidence from other countries including Kenya, South Africa, and Malawi supports the effectiveness of DHIS-2 in improving reporting rates and tracking health indicators. For instance, both Kenya and South Africa have reported improved reporting rates and effective tracking of health indicators following the adoption of DHIS-2 [14,16–18]. In Malawi, a mid-term review of the performance of the DHIS-2 system recognised it as one of the best in the continent [17]. Similarly, a study in Uganda [18] highlighted the challenges inherent with the paper-based health management information system and the positive outcomes achieved through the implementation of an online alternative.

Despite the evidence that an enhanced, standardised health reporting system plays a pivotal role in improving healthcare systems, many LMICs, particularly those in sub-Saharan Africa, still rely on paper-based data collection and storage systems for health reporting, resulting in the production of incomplete and inaccurate reports [5-7]. Our findings suggest that this pervasive challenge can be overcome via a structured approach, including clearly defining indicators to be captured, integrating them into DHIS-2, building the capacity of frontline staff and continuously supervising and coaching them to capture and enter the data. Indeed, our experience highlights the significance and advantages of DHIS-2 in optimising reporting timeliness and enhancing the overall effectiveness of decision-making processes and resource allocation for essential public health interventions such as medical oxygen. Furthermore, our experience demonstrates that the integration of oxygen indicators into DHIS-2 significantly reinforces our commitment to improving the overall quality of healthcare services related to hypoxaemia management. By capturing and monitoring hypoxaemia management and medical oxygen use data within a standardised, centralised system, we can easily identify areas needing improvement and implement evidence-based strategies to enhance patient care.

With sustained support and investment in integrating relevant indicators into DHIS-2, countries can foster more efficient, data-driven healthcare systems, ultimately improving health outcomes for their populations. The lessons we gained here hold significant value, as they not only provide insights for Cameroon, but also serve to inform future implementations of similar systems in other sub-Saharan countries. The identification and selection of key indicators for effective disease management, coupled with the active engagement of multiple stakeholders throughout the identification and implementation process, are crucial steps that contribute to the success of such initiatives and effectively address any reporting challenges that may arise. In addition to indicator selection and stakeholder engagement, it is vital to recognise the significance of training personnel at the operational level to guarantee accurate reporting and timely submission of data to the regional and, ultimately, the central level. This capacity building is essential in maximising the benefits associated with integrating variables into DHIS-2 that must be captured at the facility level. However, the process does not end with initial training alone, as they should be supplemented by continuous capacity-building initiatives and supportive supervision, particularly if they target district and facility personnel, wherein they can serve as mechanisms for providing ongoing training, support, and guidance to this cadre of personnel, enabling them to consistently maintain accurate and reliable data, which, in turn, would inform regional and national level monitoring and planning efforts.

While the use of the DHIS-2 system to monitor hypoxaemia diagnosis and management and medical oxygen use highlighted its considerable potential in improving patient outcomes, we identified several challenges that may hinder its effectiveness and sustainability. First, there was limited availability of staff at the operational level (district and facility level) trained on the use of the DHIS-2 system. This gap in human resources can critically undermine the potential for effective implementation and utilisation of the system. Furthermore, many healthcare facilities in Cameroon are constrained by the lack of essential hardware, such as computers and mobile devices required for data entry and reporting and the overall effective use of the DHIS-2 platform [19]. Internet connectivity also remains a considerable barrier, with many rural and remote areas experiencing poor internet connectivity that may hinder real-time reporting capabilities and delay data transmission [19]. These challenges, which have also been reported in other settings [20], highlight the need for a long-term strategy that considers the necessary resources for the sustainability of the DHIS-2 system in Cameroon, especially when it comes to infrastructure, training, and system maintenance [19,21].

Although our study provides valuable insights into the potential of integrating oxygen indications into DHIS2 on hypoxia case management, certain limitations must be acknowledged. We focussed on the completeness of data on hypoxaemia diagnosis/management and medical oxygen use within monthly health facility reports from DHIS-2 following the integration of indicators for medical oxygen into the system. This implies that we failed to capture information on other data quality metrics such as incoherencies and reporting timeliness, which could have provided a broader understanding of the project’s impact. Despite this limitation, we generated seven key lessons that could inform future work in this critical public health area, which we strongly believe could be leveraged to replicate similar initiatives in other LMICs akin to Cameroon:

1. To be successfully integrated into a national health information management system, oxygen indicators first need to be fully defined, after which multiple stakeholders – such as healthcare providers, district managers, and policymakers – should be engaged in their validation and implementation. This would ensure that the indicators are relevant and supported across all levels of the health system.
2. To minimise reporting delays, healthcare personnel should be trained ahead of time. This proactive approach enhances their understanding of the data entry process and the importance of accurate reporting.
3. The benefits of integrating hypoxaemia management variables into DHIS-2 can be maximised only if lower-level health facilities collect accurate data and enter it promptly for use by higher tiers of the health system for decision-making.
4. Continuous supportive supervision and mentoring are vital for building the capacity of health facilities to collect and report high-quality data to inform decision-making.
5. Encouraging a collaborative environment among healthcare workers, supervisors, and stakeholders can foster a shared commitment to improving data quality and reporting practices, leading to the development of best practices and innovative solutions to common challenges.
6. Flexibility in adapting strategies is crucial. Each region may face unique challenges, highlighting the importance of tailoring approaches to local contexts.

## CONCLUSIONS

The integration of indicators on hypoxaemia management and medical oxygen use into the DHIS-2 and the training of regional- and district-level personnel on the capture and reporting of these indicators yielded positive results, resulting in an almost 50 percentage point increase in the reporting of hypoxaemia diagnosis and treatment just within half a year of implementation. This positive result showcases the importance of a standardised, centralised, digital reporting system in streamlining the reporting process and promoting timely decision-making around key implementation and operational challenges. By driving the integration of these indicators into DHIS-2, we have provided the Cameroonian MoPH with a sustainable tool to track hypoxaemia management and medical oxygen use continuously and effectively which can now be used to make informed decisions on resource allocation and interventions for medical oxygen in order to improve patient outcomes.
